# Establishing Boundaries: The Relationship That Exists between Intestinal Epithelial Cells and Gut-Dwelling Bacteria

**DOI:** 10.3390/microorganisms7120663

**Published:** 2019-12-09

**Authors:** Amy A. O’Callaghan, Sinéad C. Corr

**Affiliations:** Department of Microbiology, School of Genetics and Microbiology, Moyne Institute of Preventative Medicine, Trinity College Dublin, 2 Dublin, Ireland; ocallaa4@tcd.ie

**Keywords:** intestinal epithelial cells, pathogen recognition receptors, tight junctions, barrier integrity, pathogens, probiotics, host–microbe interactions

## Abstract

The human gastrointestinal (GI) tract is a highly complex organ in which various dynamic physiological processes are tightly coordinated while interacting with a complex community of microorganisms. Within the GI tract, intestinal epithelial cells (IECs) create a structural interface that separates the intestinal lumen from the underlying lamina propria. In the lumen, gut-dwelling microbes play an essential role in maintaining gut homeostasis and functionality. Whether commensal or pathogenic, their interaction with IECs is inevitable. IECs and myeloid immune cells express an array of pathogen recognition receptors (PRRs) that define the interaction of both pathogenic and beneficial bacteria with the intestinal mucosa and mount appropriate responses including induction of barrier-related factors which enhance the integrity of the epithelial barrier. Indeed, the integrity of this barrier and induction of appropriate immune responses is critical to health status, with defects in this barrier and over-activation of immune cells by invading microbes contributing to development of a range of inflammatory and infectious diseases. This review describes the complexity of the GI tract and its interactions with gut bacteria.

## 1. Introduction

The human body is designed to have several levels of complexity and organization; cells, tissues, organs, and organ systems. Functioning as one, human organ systems supply all the cells of the human body with essential biological materials required to survive and thrive, as well as removing any biological waste to ensure homeostasis is maintained. The digestive system has three main functions: digestion of food, absorption of nutrients, and elimination of solid food waste. These functions are enabled through coordinated action of the gastrointestinal (GI) tract (e.g., mouth, esophagus, stomach, small intestine, large intestine) and accessory organs (pancreas, gall bladder, and liver). To describe the digestive system in more detail, the GI tract comprises a muscular tube-like system of organs split into two main sections; the upper and lower GI tract. The upper GI tract consists of the mouth, esophagus, stomach, and duodenum. The lower GI tract encompasses the vast majority of the mucosal surface and describes the ileum, jejunum, colon, cecum, rectum, and the anus. Both the upper and lower GI tract are major sites of digestion and absorption of nutrients from ingested food [1], as well as being primed for defense against infection. The cross-sectional structure of the GI tract is generally compartmentalized by the mesentery, serosa, muscularis, submucosa, lamina propria, epithelium, and lumen. Major arteries, veins, lymphatics, and nerve fibers enter and exit the tissue through the mesentery. It also encapsulates the mesenteric lymph nodes, which are draining lymph nodes of the intestine. The intestinal serosa is a smooth membrane made up of a single layer of cells that secrete serous fluid, a lubricating fluid. The muscular layer provides the physical force for the mechanical digestion of food including movement of the bolus downward, churning in the stomach and movement of ingested food through the intestines and elimination of waste through the rectum. The submucosa consists of a layer of connective tissue joining the muscular layer with the innermost mucosa. Blood vessels are vastly rich in the submucosa, as are various types of nervous tissue. Due to this extensive system of neurological activity, it is highly understood that gut physiology may have a significant effect on human behavior [2]. The mucosa is the innermost layer, curved around the open gut lumen and is split into three sublayers—the muscularis mucosae, the lamina propria, and the epithelium. The lamina propria is home to a colossal range of immune cells, considering that the GI tract is a major entry point for exogenous pathogens. The intestinal epithelium is the largest of the body’s mucosal surfaces, covering ~400 m^2^ of surface area with a single, continuous layer of cells organized into crypts and villi, acting as an important semipermeable barrier between microbes and the underlying host’s innate immune system. The gut lumen is inhabited by a tremendous number of microorganisms, termed the gut microbiota, with a microbial load of 10^11^ bacteria/mL content in the colon [3]. The GI tract has two distinct microbial ecosystems, the luminal and the mucosal microbiota [4]. In the luminal compartment, over 90% of bacteria belong to *Firmicutes* and *Bacteroidetes*, with minor phyla including *Actinobacter*, *Proteobacteria,* and *Verrucomicrobia*. Differences occur in the mucosal layer, where the number and diversity of the microbiota are considerably lower. The composition of the microbiota is clearly different, as *Firmicutes* are generally higher in abundance compared to *Bacteriodetes* in both humans [5] and mice [6]. Importantly, the density and composition of the microbiome change along the GI tract, with major populations being selected for by nutritional availability and the functions performed at various locations. The upper GI tract or small intestine typically has high levels of acids (e.g., bile acids (BAs)), oxygen and antimicrobials, and a short transit time. These properties limit bacterial growth such that only rapidly proliferating, facultative anaerobes with strong epithelial adherence abilities are thought to survive [7]. In contrast, colonic conditions support a dense and diverse community of bacteria, mainly anaerobes that are capable of fermenting complex carbohydrates that are undigested in the SI. Interestingly, only 5% of secreted BAs reach the colon and are faced with several microbial-mediated biotransformations. Indeed, a disrupted gut microbiota including reduced bile metabolizing bacteria significantly impairs BA metabolism and, subsequently, the host metabolic pathways regulated by BA signaling, in turn affecting glucose and cholesterol homeostasis, as well as immune states. Indeed, inflammatory conditions have been linked to gut dysbiosis and altered BA profiles in humans [8]. Thus, the relationship that exists between man and microbe is evolutionary harmonizing, where a pleiotropic network of immune, metabolic, and trophic functions prevails. 

## 2. Cells of the Intestine and Their Interactions with Microbes

Intestinal epithelial cells (IECs) exist as a layer of columned cells that line the luminal surface of intestinal epithelium, generating a functional barrier to protect the intestinal mucosa from commensals or invading pathogenic microorganisms. IECs are continually generated and replaced every 4–5 days through a process of renewal and migration. Stem cells residing in the crypts are responsible for the high throughput of surface residing cells and are indispensable for intestinal homeostasis and preventing bacterial invasion. Older IECs undergo apoptosis and are shed into the intestinal lumen, with the aid of passing ingested material. Several cell types are present in the intestinal epithelium, including enterocytes, Paneth cells, goblet cells, and enteroendocrine cells, all contributing to the formation of physical as well as chemical barriers against microbial threats.

### 2.1. Enterocytes

Enterocytes are hyperpolarized epithelial cells that are joined together by tight junctions (TJs). Efficient protein sorting and addressing are crucial to the establishment and maintenance of cell polarity, on which the intestinal epithelial barrier highly depends. The apical surfaces of enterocytes are covered by rigid, closely placed microvilli [9], the tips of which contain large, negatively charged, integral membrane mucin-like glycoproteins that form a continuous, filamentous brush border glycocalyx [10]. The glycocalyx layer prevents direct contact of microbes, viruses, macromolecular aggregates, and particles with the microvillus membrane [11]. It has been speculated that enterocytes act as antigen-presenting cells and can regulate T cell responses in the intestinal mucosa [12]. Enterocytes express surface receptors that initiate engulfment of antigens. From here, pathogenic antigens are processed and are presented to the underlying immune system on major histocompatibility complexes I or II [13]. Without infection or inflammation, enterocytes downregulate T cell responses. Thus, insights in to how enterocytes engulf antigens and deliver these to the underlying gut-associated lymphoid tissue (GALT) in order for an appropriate immune response to be mounted against luminal antigens can be of great importance for the development of oral vaccines, or the understanding of intestinal disorders in which tolerance induction against food antigens or commensals is disturbed, like food allergy, celiac and inflammatory bowel disease (IBD). 

### 2.2. Goblet Cells

An important physical barrier in the GI tract includes the mucosal layer that overlays IECs, creating the first line of defense against microbial encroachment, thus preventing aberrant immune responses directed against the gut microbiota. Goblet cells secrete highly glycosylated mucins that create a net-like layer, mainly consisting of mucin 2 (MUC2). The small intestine mucosal layer is discontinuous whereas the colon contains a mucosal layer that is separated into two layers [14]. The innermost layer is highly compact and is largely sterile whereas the outermost layer is looser and contains commensals that thrive in the lumen, considering that the colon is a microbe dense organ. These highly regenerative mucosal layers have long been considered as lubricators, easing the passage of fecal material through the intestinal channel. However, recent research has led to a greater understanding of how the gut microbiota interacts with this complex layered intestinal component. Mucus provides a niche for bacterial colonization, where distinct commensals utilize mucus as a carbon source [4]. A diverse microbiota contributes to the thickening and flourishing of mucosal layers [15]. This is supported by Szentkuti et al., with germ-free (GF) rats displaying a thinner or almost absent layer of mucus that was structurally altered when compared to conventional rats [16], outlining the influence of the gut microbiota. Changes in other factors such as dietary fiber intake result in heightened utilization of mucin-associated carbohydrates by Bacteroides species, thinning the protective inner mucin layer and reduces host resistance to infection [17]. Mucin production is stimulated through the activation of pathogen recognition receptors (PRRs) present on the surfaces of IECs. This stimulation can occur directly through recognition of lipopolysaccharide (LPS), or indirectly through secretion of pro-inflammatory cytokines. Recognition of LPS by LPS binding protein (LBP) or Toll-like receptor (TLR) 4 yields a strong pro-inflammatory response, inducing mucin gene expression [18]. Of recent, the activation of the inflammasome mediated by nucleotide-binding oligomerization domain (NOD)-like receptor (NLR) family pyrin domain-containing 6 (NLRP6), a member of the NLR family, drove the secretion of mucus from goblet cells through the promotion of autophagy [19]. Gut-dwelling microbial by-products, such as short-chain fatty acids (SCFAs), have also shown to stimulate mucus secretion from IECs [20]. There is a lack of evidence describing the interaction of probiotics with Goblet cells inferring mucin production. One study describes how mice mono-colonized with *Lactobacillus (L.) acidophilus* displayed a similar level of MUC2 expression when compared to GF mice [21]. Thus, intestinal mucus production requires an association with the complex collective community of intestinal microorganisms to ensure its full functionality. Mucin production importance is highlighted by spontaneous development of colitis and predisposition to inflammation-induced colorectal cancers, which has been observed in MUC2-deficient mice [22,23]. 

### 2.3. Paneth Cells

Intestinal epithelial barrier function is further reinforced by antimicrobial peptides (AMPs) secretion by Paneth cells, which are located at the bottom positions of small intestinal crypts. Paneth cells are only present in the small intestine, thus the expression level of AMPs in the large intestine is noncomparable with that in the small intestine [24]. These cells contain lysozymes, secretory phospholipase A2, and α-defensins collectively stored in secretory granules. A plethora of microbes inhabiting the gut luminal area interact with Paneth cells, ensuring sufficient protection from otherwise transient microorganisms. Apical stimulation of PRRs such as TLRs primed by LPS has been described by the immediate degranulation and secretion of AMPs by Paneth cells, indicating that Paneth cells directly sense LPS [25]. Gong et al. describe how the deletion of the signaling adaptor protein myeloid differentiation primary-response gene 88 (MyD88) results in decreased production of RegIIIγ, RELMβ, and RegIIIβ by intestinal epithelium, increasing susceptibility to *Salmonella enterica* serovar Typhimurium [26]. Whether these cells are able to distinguish pathogenic bacteria from commensals remains unexplored to date [27]. However, it has been argued that Paneth cells do not interact with bacterial antigens directly, but that an unknown immune cell releases the inflammatory mediator interferon gamma (IFN-γ), indirectly stimulating Paneth cell secretion of AMPs [28]. Burger et al. demonstrate how microbiota-driven IFN-γ is a central inducer of Paneth cell autophagy [29], a fundamental cellular process required for the recycling of intracellular components. Autophagy is indispensable to a variety of cellular processes, including orchestrating host defense responses. As such, crosstalk between microbes and Paneth cells protects the intestinal epithelium, particularly during intestinal inflammation. AMPs secretory levels are altered in GF mice [30], depicting how microbiota composition heavily influences the functioning of these important IECs. Cazorla et al. recently published data outlining how a probiotic cocktail of *L. casei* CRL 431 and *L. paracasei* CNCM I-1518 administered to mice increased the number of Paneth cells in the small intestine, aiding in the elimination of infectious disease [31]. Paneth cell dysfunction is associated with many diseases such as IBD, obesity, and graft-versus-host-disease (GvHD). In GvHD model mice, loss of secreted α-defensins due to a lack of healthy Paneth cells causes a shift in composition of the gut microbiota, termed dysbiosis, resulting in fatal sepsis [32,33]. 

### 2.4. Enteroendocrine Cells

Enteroendocrine cells (EECs) are located in the crypts of the GI tract and produce a variety of neuroendocrine molecules. These cells have been commonly referred to as the ‘taste’ cells of the gut due to their popularity at chemosensing; they have the capacity to sense the luminal nutrient environment of the gut. They also have the ability to control the release of molecules involved with aspects of feeding, such as appetite [34]. It is known that the gut microbiome influences the release of such molecules from EECs, having major benefits for both the microbiota and the host. Recent evidence has outlined how microbial-mediated release of these molecules may influence other types of EECs [35], depicting a complex rapport between the intestinal microbiota, gut hormone release, and host metabolism. EECs express TLRs and activation of such receptors by microbes trigger secretion of a selection of metabolically active hormones such as glucagon-like peptide 1 (GLP-1) [36], serotonin (5-HT) [37], and peptide tyrosine-tyrosine (PYY) [38]. Collectively, these hormones augment insulin [39], regulate mood, and induce satiety [40], respectively. Indigenous spore-forming bacteria from both mouse and human microbiota promote 5-HT biosynthesis from enterochromaffin cells, which supply 5-HT to the mucosa, lumen, and circulating platelets [41]. This research uncovers a role for the microbiota in regulating the brain serotonergic system, influencing host physiology. Since microbiota influences central nervous system (CNS) through various immunological pathways (e.g., TLRs), microbial dysbiosis is considered disease-promoting in many neurological disorders such as multiple sclerosis (MS), for example. Clinical studies have observed specific taxa in microbiomes observed in patients with MS. Transplantation of these bacteria from MS patients into GF mice exacerbated experimental autoimmune encephalomyelitis (EAE) via increased pro-inflammatory T cell response and weakened T regulatory (Treg) response [42]. In addition, treatment of MS by probiotic VSL3 induces enrichment of specific microbial species in intestine and inhibits peripheral monocyte-mediated inflammation [43]. EECs indeed express receptors that recognize SCFAs [44], which are produced at an elevated rate through the fermentation of fiber by gut microbiota. Through indirect contact, gut microbiota regulates hormones associated with food intake and insulin secretion [45], areas of translational interest in the field of obesity and diabetes. Not surprisingly, the gut microbiota controls the differentiation of EECs, in particular, a distinct type of EEC known to secrete PYY and GLP-1 [46]. Though EECs make up less than 1% of all IECs, it is clear that their interaction with the microbiota is essential for maintenance of host health, potentially functioning as an individual endocrine organ and using metabolites and hormones as key messengers to interact with other organs, such as the brain. 

### 2.5. M Cells

Defined as microfold or membranous cells, M cells are unique IECs responsible for immune sensing bacteria sequestering in the lumen. Despite there being relatively few numbers within the intestinal epithelium, M cells play a pivotal role in the initiation of the immune response, continuously sampling the lumen of the small intestine and transporting antigens to underlying dendritic cells (DCs) [47]. M cells express various PRRs that initiate phagocytosis once pathogens are detected. Using Glycoprotein 2 (GP2) knockout mice and mutated bacteria, Hase et al. revealed how GP2 acts as a receptor for Type I pili on a subset of Gram-negative enterobacilli such as *Escherichia coli* and *Salmonella enterica* [48]. Thus, GP2 is essential for immunosurveillance at mucosal surfaces by initiating efficient mucosal immune responses against commensal as well as pathogenic bacteria. Cellular prion protein (PrPc) is a ubiquitously expressed glycosylphosphatidylinositol (GPI-) anchored protein which is highly expressed on the apical surface of M cells [49]. PrPc is known to interact with pathogens that contain the well conserved, immunogenic surface protein heat shock protein 60 (HSP60) [50]. M cells express immunoglobin A (IgA) receptors on their apical surface, transporting secreted IgA (sIgA)-bound antigens into Peyer’s patches. sIgA acts by flagging commensal bacteria with a propensity to elicit an outraged host immune response and induce colitis, leading to intestinal pathology [51]. It is evident that without M cells an adequate immune response would not occur, facilitating pathogenic infection and intestinal inflammation. 

### 2.6. Tuft Cells 

Tuft cells are another unique epithelial subset which is endowed with the ability to detect luminal factors, including microbe- and parasite-derived products. Always considered enigmatic, it was suggested that these cells have a sensory function, supported by the expression of several components of the taste receptor signaling cascade [52]. Cheng et al. describe a subpopulation of tuft cells that contain 5-HT and are in close anatomic contact with enterochromaffin cells, advancing knowledge on these sensory cells and their role in the regulation of gut 5-HT levels [53]. However, recent publications describe how mucosal tuft cells constitutively express interleukin-25 (IL-25) and that these cells increase significantly in small intestinal epithelia after infection with helminths, assisting infection resistance in the host and a reproductive niche for luminal pathosymbionts [54]. This data is supported by other publications that have shown how the microbial- and parasite-derived metabolite succinate activates succinate receptors (SUCNR1) on tuft cells, initiating and flourishing a type 2 immune response [55]. Despite these recent advances, many fundamental questions about tuft cells in immunity remain to be answered. 

## 3. Intestinal Epithelial Barrier Integrity Influenced by Microbes

A necessary component of the intestinal epithelial barrier is the intercellular junctional complexes which are fundamental in the maintenance of intestinal barrier integrity. TJs form a strong seal between neighboring cells near the apical surface, preventing paracellular diffusion of microorganisms and other antigens across the epithelium. The functional integrity of the intestinal epithelium allows a balance to be maintained between the absorption of nutrients and the sequestration of inflammatory stimuli and is a key protective mechanism against diseases of the bowel. Other junctional complexes include gap junctions, adherens junctions, and desmosomes [56]. Adherens junctions lie beneath TJs and are involved in cell to cell adhesion and intracellular signaling. Both of these junctional complexes interact with actin cytoskeleton of the cell. Desmosomes and gap junctions engage in cell to cell adhesion and intracellular communication, respectively. Evidently, many pathogens exploit such physical barriers to either evade cells and/or tissues or to promote signaling responses to facilitate tissue invasion. The majority of bacteria that challenge TJs are ingested through foods we eat or water that we drink. Many pathogens impair junctional structures indirectly by activation of signaling cascades of host cells. Alternatively, pathogens can provoke inflammatory outbreaks, which damage TJs. It is well known that a ‘leaky’ gut is the main factor contributing to the pathogenesis of IBD. Table 1 summarizes the effects of pathogenic bacteria directly interacting with TJs and the outcome of barrier integrity (Table 1). 

On the other hand, commensal bacteria and probiotics have been shown to promote intestinal barrier integrity both *in vitro* and *in vivo*, restoring the balance of the gut microbial ecosystem, where pathogenic infections could be somewhat controlled. Probiotics are defined as live microorganisms which when administered in adequate numbers confer a health benefit on the host [67]. Published data has shown that probiotics enhance the intestinal barrier in mouse models of colitis [68] and reduce intestinal permeability in human patients with Crohn’s disease [69]. The capacity of probiotics to interact with IECs has been demonstrated, with one group describing how *L. casei* and *L. paracasei* adhere to IECs through TLRs, mediating immune stimulation [70]. The exact mechanism of how probiotics act in the intestine remains unknown; however, expanding the literature on microbe–cellular interaction is becoming more readily available but much more work is required to fully understand the complexities of the relationship between probiotics and gut health. Table 2 summarizes recently published data describing the effects of various probiotic bacterial strains directly interacting with TJs during intestinal inflammation. 

It is important to highlight that metabolism is a crucial regulator of TJ formation. Zhang et al. demonstrated how activation of 5′ AMP-activated protein kinase (AMPK) boosted endogenous Ca^2+^ levels in kidney cells, driving TJ formation [71]. Additionally, hypoxia-inducible factor (HIF-1α) expression is critical for SCFA regulation of intercellular permeability [72], as HIF-1α is known to promote intestinal barrier factors and reduce apoptosis, resolving inflammation [73]. Interestingly, AMPK activation has been shown to stabilize HIF-1α and prevents glycolysis from occurring, the Warburg effect, implicating an important role for butyrate in modulating glycolysis [74]. Finally, Peng et al. demonstrated that butyrate, a known activator of AMPK, modulates TJ formation through AMPK regulation [75]. Thus, microbial-derived products, butyrate, in particular, contribute to the formation of TJs. Recent studies confirm how a dysbiotic gut microbiota negatively influences AMPK signaling pathway, where *Prevotella* relative abundance triumphs in comparison to SCFA-producing bacterial populations [76]. To support published AMPK data to date, Olivier et al. recently demonstrated how AMPK activation in Caco-2 cells ensures a better recovery of epithelial barrier function following injury [77]. Here we see the symbiotic relationship between host and microbe succeed, where microbial by-products promote barrier integrity, ensuring that microbial communities remain sequestered in a healthy gut lumen.

## 4. Conclusions 

**Figure 1:**
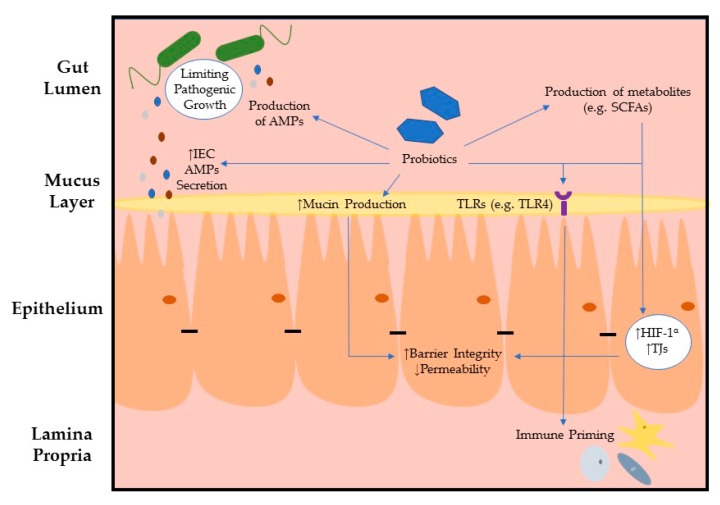
Interactions between gut commensals and the intestinal epithelium reinforces barrier defence.

Certain commensal gut colonizers reinforce the host epithelium, producing immunoregulatory effects indirectly, i.e., through the secretion of by-products such as SCFAs, or directly through interaction with PRRs expressed on the apical surface of IECs (Figure 1). In contrast, pathobiont bacteria manipulate various components that contribute to gut health directly by interacting with TJs or indirectly, through activation of the host’s immune response. This results in the development of dysbiosis (a shift in the composition of resident intestinal microbes) and prolongs inflammation following infection. TJs are associated with physical intestinal barrier function, standing at the interface of host–microbe interactions. Evasion of TJs by pathogens profoundly impacts host health, while consumption of specific probiotics may represent a powerful tool to re-establish gut homeostasis and promote gut health by targeting TJs. Collectively, the studies described in this review highlight the diverse and multifaceted roles of IECs in conjunction with intestinal microbiota interactions in the exacerbation of inflammation and persistence of infection or in the maintenance of intestinal homeostasis, and the complexity of the relationship between these two different scenarios. This review reiterates how microbial and mammalian cells interact intimately together during health and disease.

## Figures and Tables

**Table 1:** Pathogenic intestinal disease progression through tight junctional manipulation.

Bacteria	Mechanism of Invasion	Effects on TJs	Reference
*Escherichia coli AEEC*	Type-three secretion system (TSS3) of effector proteins; EspF [57], EspG [58], and MAP [59]	Ultrastructural alteration of TJ strands	
		Indirect interaction with TJs but with actin-associated proteins	[60]
		Redistribution of TJs	[60,61]
*Salmonella typhimurium*	Salmonella pathogenicity island (SPI1) injected into host cell using T3SS SPI1 effectors; SopB, SopE, SopE2, and SipA [62]	Decrease in zonula-1 (ZO-1) expression and occludin phosphorylation	[63]
*Shigella flexneria*	T3SS injection of effector proteins; SepA [63]	ZO-1, claudin-1, and occludin are all phosphorylated Decrease in negative-inhibition of actin-remodeling	[64]
*Listeria monocytogenes*	T3SS injection of effector protein; InlC [65]	Breakdown of barrier integrity	
*Helicobacter pylori*	T4SS delivery of effector protein; CagA [66]	Incomplete assembly of TJs	

**Table 2:** Probiotic strains improving intestinal disease through modulation of tight junctions (TJs).

Probiotic Bacteria	Intestinal Disease	Effects on TJs	Reference
*L. plantarum* (ATCC 10241) & *L. rhamnosus* (ATCC 53103)	Necrotizing enterocolitis (NEC)	TJ structure reinforced and secured *in vitro* Probiotic acting on TJs *in vivo* in a similar manner as seen *in vitro*, however, a varied gut microbiota determines efficacy of probiotic	[78]
*L. reuteri* (LR1)	Enteric pathogen infection using ETEC K88	Enhanced expression of ZO-1 and Occludin	[79]
*L. rhamnosus* (GG) & *L. reuteri* (ZJ617)	LPS-induced barrier dysfunction	Enhanced expression of Occludin and Claudin 3	[80]
*Bacillus subtilis* (29784)	Pro-inflammatory cytokine induced intestinal inflammation	Enhanced expression of ZO-1, Occludin, and Claudin-1	[81]
*Bifidobacterium*, *L. acidophilus* & *Enterococcus*	Dextran sodium sulfate (DSS)- induced colitis	Increased expression of TJs and microstructures overall improved	[82]
